# Application of Carbon Nanoparticles in Neck Dissection of Clinically Node-Negative Papillary Thyroid Carcinoma

**DOI:** 10.1155/2021/6693585

**Published:** 2021-04-21

**Authors:** Zhongyan Chen, Zhiming Zhong, Guoqing Chen, Yun Feng

**Affiliations:** ^1^Department of Otolaryngology, China-Japan Friendship Hospital, Beijing 100029, China; ^2^Department of Otolaryngology, Jianou Municipal Hospital, Fujian 353100, China; ^3^Department of Otolaryngology, Jinnan Branch of Jinjiang Municipal Hospital, 362241 Fujian, China

## Abstract

**Purpose:**

The purpose of this retrospective study was to evaluate the advantages of carbon nanoparticles in neck dissection and to conclude its application in the treatment of clinically node-negative papillary thyroid carcinoma (CN0PTC).

**Methods:**

As a retrospective cohort study, we divided the enrolled patients into two groups, the carbon nanoparticle (CN) group and the control group according to the usage of CN. In the CN group, CN was applied to reveal drainage lymph nodes and the picked LNs were sent for fast frozen testing. If metastasis exits, modified radical lateral lymph node dissection (LLND) was performed. For both groups, prophylactic central lymph node dissection was routinely done. Finally, the demographic information, tumor characteristics, postoperative pathological results, and laboratory data were collected for analysis.

**Results:**

A total of 61 CN0PTC were enrolled in this study, 33 in the CN group and 28 in the control group. The black-stained rate for CN was 29/40 (72.5%) with a positive prediction rate of 34.5%. The mainly black-stained region in the lateral neck was level III and possesses the highest lymph node ratio (17.5%). The metastasis that occurred in level VI was 30% and 11.8% in the CN and control groups, respectively (*p* = 0.058). During the available follow-up, no one showed recurrence. Statistical analysis showed that the CN suspension can significantly reduce the risk of damage to the parathyroid gland (*p* = 0.001 for hypocalcemia, <0.05; *p* = 0.047 for hypoparathyroidism, <0.05).

**Conclusion:**

The lateral neck metastasis in patients with papillary thyroid microcarcinoma in clinical stage cT1aN0 is not rare. CN can help surgeons to distinguish the real person who actually needs LLND. In prophylactic CLND, CN acts as a tracer which makes the parathyroid gland more identifiable and avoids risks of injuries to nerves and glands.

## 1. Introduction

With the development of ultrasonography, especially with the coming out of high-resolution machines, the incidence of thyroid cancer in China is dramatically increasing in recent years [1]. Over 90% of thyroid malignancies are differentiated thyroid cancer (DTC), most of which are papillary thyroid carcinoma (PTC) [2]. PTC is insensitive to either chemotherapy or radiotherapy and metastasis via mainly the lymphatic system. Up to now, surgery including thyroidectomy plus neck dissection is the best means of treatment. Importantly, the surgical strategy about lymph nodes is an essential issue that concerns every head and neck surgeon. In literature, a series of meta-analyses and clinical articles have discussed the outcomes of pCLND (prophylactic central lymph node dissection). A recently published retrospective study analyzing 399 patients to explore the role of prophylactic central lymph node dissection (pCLND) showed that pCLND can improve disease-free survival in patients with intermediate and high risk of disease recurrence [3]. Indeed, pCLND is believed to reduce locoregional recurrence rate and provide pathological evidence for the adjuvant radioiodine (RAI) treatment [4]. Nevertheless, some researchers thought that during the CLND, the protection of parathyroid glands and recurrent laryngeal nerve (RLN) injury plays a vital role due to its otherwise injury-causing poor life quality. Additionally, the role of lymph node metastases on prognosis and mortality is some kind doubtful. Gambardella et al. considered that pCLND has no positive influence on recurrence rate but brings higher postoperative complications [5].

Besides pCLND, there have been limited studies focusing on pLLND. Therefore, after a long period of academic discussion, prophylactic central (pCLND) and even lateral neck dissections (pLLND) in patients with clinically negative lymph nodes (CN0PTC) are still controversial. It is reported that the event of skip metastasis (lateral neck LN metastases without central LN metastasis) is not rare [6]. Considering all the above, we believed that importance should be attached to both pCLND and pLLND, and improving the safety and efficacy of pLLND is vital so that patients can get benefits from neck dissection while life quality will not be disturbed.

Since 2015, our center has been using carbon nanoparticle (CN) suspension to trace lymph nodes to reduce the occurrence of LND complications and lower the risk of disease recurrence. CN is made of particles with 150 nm diameter which allows CN to enter the lymphatic capillaries (the intercellular space of lymphatic capillary cells is 120–500 nm), but not the blood capillaries (the intercellular space of blood capillary cells is 20–50 nm). Taking use of those properties, CN can detect draining lymph nodes which are of importance in the surgical decision-making. Although certain articles about CN in thyroid cancer have been published, we wonder how the CN suspension was applied in PTC patients and the benefits it brings to the real-world condition. In order to conclude the five years' experience in the usage of CN, we conducted this retrospective cohort study based on the assumption that CN suspension can help the detection of occult lateral neck metastasis and significantly reduce the occurrence of associated complications. We hoped this work can uncover the application of CN in prophylactic neck dissection in CN0PTC patients and explore the influence of CN on the occurrence of hypocalcemia and hypoparathyroidism.

## 2. Method

A total of 61 patients diagnosed as CN0PTC from August 2015 to December 2019 at the Department of Otorhinolaryngology-Head and Neck Surgery of our center were collected retrospectively. The study was approved by the Institutional Review Board of the China-Japan Friendship Hospital, Beijing, China. Informed written consent was obtained from the enrolled patients.

### 2.1. Inclusion Criteria

1. Patients with fine needle aspiration cytology- (FNAC-) proven PTC without suspicious lymph nodes in ultrasound (CN0PTC) were recruited. No history of head and neck surgery or radiation
2. All patients had documentation of normal vocal cord mobility by preoperative laryngoscopy
3. A preoperative neck ultrasound and neck CT scan showed no evidence of metastasis occurring in central and lateral cervical compartments (CN0PTC). Both ultrasound and neck CT scan were confirmed by two experienced specialists
4. Patients were included in the CN group or control group according to the usage of CNs or not

### 2.2. Exclusion Criteria

1. Patients in whom FNA had confirmed lateral neck lymph node metastases were excluded from this study
2. Patients with aggressive histologic variants (columnar tall/tall cell, diffuse sclerosing, and insular types) were excluded
3. Patients were also excluded if the frozen pathology examination during operation confirmed benign tumor or any other malignant tumor, such as medullary thyroid cancer and thyroid lymphoma
4. Patients younger than 18 years, pregnant, breastfeeding, unable to participate in a postoperative follow-up, or unable to give informed consent were excluded

### 2.3. Procedure

#### 2.3.1. Surgical Technique

All surgical procedures were performed by the same surgeon (Prof. Yun Feng) with high experience in thyroid surgery. General anesthesia was given to all patients. The patients were placed in the supine position. A cervical collar curved incision was made.

**Figure 1:**
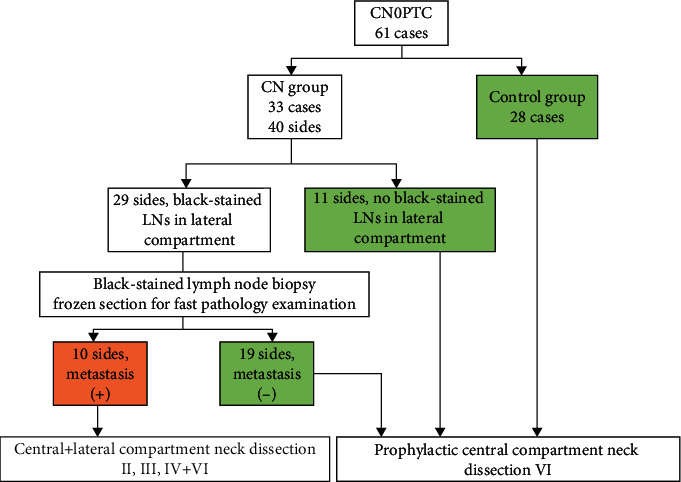
A flow diagram used to describe the therapeutic regimen for CN and control group, CN0PTC, and papillary thyroid carcinoma without suspicious lymph nodes in ultrasound. LN: lymph node.

A 1 ml syringe was used for CN suspension injecting into the thyroid gland. Several injecting spots were randomized and uniformly distributed surrounding the tumor. For each spot, 0.05 ml-0.1 ml CN suspension was slowly injected after aspirate to ensure no flashback of blood. Then, manual pressure was given using a wet gauze sponge for 15 min to promote the diffusion of CN. We could see the CNs gradually diffused from the injecting spots to the surrounding area, until the entire thyroid lobe was uniformly stained black. Next, the region of level III was first investigated assisted by endoscopy to find black-stained LNs. Then, levels II, IV, and VI were explored in turn. Unilateral lobectomy plus isthmusectomy was performed for unilateral lesion, and a total thyroidectomy should be considered for patients with bilateral nodularity and other reasonable indication (such as combined with hyperthyroidism). The black-stained LNs in the lateral and center compartments were picked for the intraoperative frozen section. In the cases where metastasis was detected in black-stained LN upon frozen biopsy, an immediate modified radical LLND along the jugular and carotid vessels (including II, III, and IV regions) plus level VI should be done. If there is no evidence of LN metastasis in the lateral compartment, therapeutic lymph node dissection was only performed in level VI (central compartment). Due to the high occurrence of central compartment metastasis, we conducted prophylactic CLND for all 61 patients no matter if CN or control group (see Figure 1).

The boundaries of the central compartment of the neck (level VI) include the hyoid bone superiorly, right and left carotid arteries laterally, and the plane at the level of the innominate artery inferiorly. This includes the pretracheal, prelaryngeal, and paratracheal lymph nodes. Levels II, III, and IV represent the lateral compartment, bordered inferiorly by the clavicle, superiorly by the digastric muscle, anteriorly by the sternohyoideus, and posteriorly by the sternocleidomastoid (SCM) muscle [7]. Level V which is referred to as the posterior triangle group includes the anterior border of the trapezius muscle laterally, the posterior border of the SCM medially, and the clavicle inferiorly and is divided into two sublevels, VA and VB using the horizontal plane that corresponds to the inferior border of the cricoid cartilage.

#### 2.3.2. Data Collection

Demographic information about age, gender, history of abnormal thyroid function, cytological results, BRAF gene mutation, intraoperative bleeding, and surgery duration was collected. Preoperative FT3, FT4, TSH, Tg, and anti-Tg antibody levels were also evaluated. Levels of calcium and parathyroid hormone, along with any complications including hemorrhage, wound infection, seroma, hypocalcemia, and vocal cord palsy, were registered postoperatively and at the follow-up after one month, six months, and 12 months. Postoperative hypocalcemia is defined as lower than 2.0 mmol/l and 12 pg/ml for hypoparathyroidism. The lymph node yield was defined as the number of lymph nodes harvested after lymphadenectomy, and the lymph node ratio was defined as the ratio between metastatic lymph nodes and total lymph nodes retrieved, calculated only in patients with metastatic lymph nodes.

#### 2.3.3. Statistical Analysis

SPSS 22.0 software (Chicago, IL, USA) was used for statistical analyses. The continuous variables were expressed as the means ± standard deviations (SD). Comparison between groups was done using *chi-square test* and *ttest*. *p* values < 0.05 were considered statistically significant.

## 3. Results

A total of 61 CN0PTC were enrolled in this study, 33 in the CN group (8 were men and 25 were women) and 28 in the control group (4 were men and 24 were women). Age, sex, BRAF mutation results, primary tumor locations and size, tumor extent, number of tumors, thyroid procedures performed, black-stained LN location and numbers, LN pathological results, and other characteristics of these patients are shown in Table 1. There was no significant difference between the two groups in gender, age distribution, tumor size and location, laterality, multinodules, and BRAF mutation (*p* < 0.05). In both groups, no patients provided family history of malignant thyroid tumor, and 2 in the CN group had hyperthyroidism under stable control of drugs. No permanent hypocalcemia and hypoparathyroidism occurred in either the CN group or the control group. As for transient hypocalcemia and hypoparathyroidism, CN can significantly avoid parathyroid injuries (*p* < 0.05, see data in Table 1). Other complications including hemorrhage, wound infection, and seroma are listed in Table 1. Only 2 patients in the control group showed unilateral vocal cord palsy and recovered after three months of medical therapy. The occurrence of complications showed no significant difference between the two groups.

**Figure 2:**
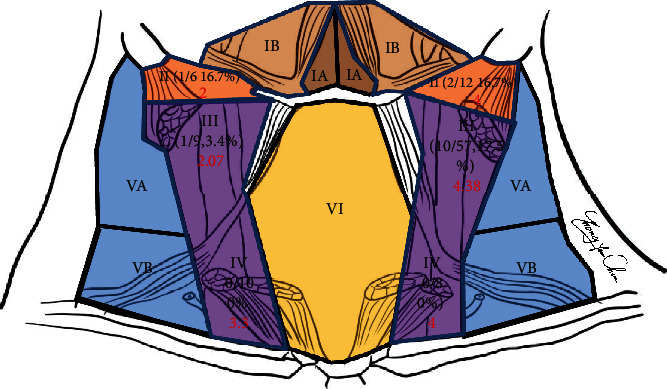
The distribution of the black-stained lymph nodes in different areas. Of these, level III has the highest possibility of detection of black-stained LN, while the metastasis in LN mostly occurs in levels VI, III, and II, and seldom involves levels V and I which is consistent with the literature. The mean lymph node yield in different areas was expressed in red.

In the CN group, seven patients were confirmed to be suffering from bilateral thyroid cancer. Finally, a total of 40 sides were collected for analysis, in which 11 sides showed no black-stained LN in the lateral compartment after CN injection; as a result, the black-stained rate of this study was 29/40 (72.5%). Ten sides (10/29, 34.5%) in which lateral compartment metastasis was revealed on frozen sections underwent immediate LLND. Of these, five sides also suffered from central compartment metastasis. The central compartment (VI) LN metastatic rate was 30% (12/40). The black-stained LN distribution in different neck levels is shown in Figure 2. In conclusion, the positive prediction rate of CN suspension was 34.5% (10/29). Distribution of black-stained LNs in the lateral compartment was shown in Table 2. In the lateral neck area, 142 sentinel LNs detected by CN suspension in 33 patients were located in the ipsilateral lateral neck compartment. The most common location of lack of stained LNs was level III (Figure 2). Black-stained level VI LNs were observed in 5 sides with 3 persons was confirmed as positive for cancer metastasis.

As for the control group, 4 sides were proofed with central compartment metastasis (4/33, 11.8%). Parathyroid tissue was spotted in the dissected soft tissue from level VI in 3 patients. 17 patients show transient hypocalcemia while 14 patients suffered from hypoparathyroidism after surgery.

## 4. Discussion

This study investigated the application of CN suspension in CN0PTC by retrospectively analyzing two cohorts: the CN group and the control group. We found that the black-stained rate of CN is 72.5% with a positive prediction rate of 34.5%, that is, the occult lateral compartment LN metastasis possibility is 35%. The black-stained LNs were mainly located in level III, then levels II and IV. Cancer primarily influences the level III and II LNs in the lateral compartment. We compared the occurrence rate of hypocalcemia and hypoparathyroidism. It showed that CN promotes the identification of the parathyroid gland when the CLND was performed and mitigates injuries to the blood supply of the parathyroid gland. Moreover, with the application of CN, we can do pLLND via a form of picking berries then undergo modified radical LLND after positive frozen pathological feedback. Zhou et al. analyzed a total of 127 patients who were diagnosed with unilateral papillary thyroid microcarcinoma (clinical stage cT1aN0M0) who underwent primary thyroid surgery (hemi- or total thyroidectomy with ipsilateral central lymph node dissection (CLND) plus lateral lymph node dissection (LLND) including levels III and IV). They found that the total metastasis rate of the lateral lymph node was 21.26% (27/127), mainly located in level III [8]. Others also reported similar data [9].

The primary therapy for differentiated (papillary and follicular) thyroid cancer is surgery including total (or near-total) thyroidectomy and unilateral lobectomy and LND. For CN0PTC, LN metastasis occurs mostly in the central compartments while the lateral compartment LNs are also commonly involved. However, most research results primarily focused on the stained nodes in the central compartment so that the application of CN in CN0PTC lateral LND was rarely reported, and whether CN can improve the detection rate of metastatic lateral compartment nodes is still uncertain. It is believed that lateral LN metastasis was associated with tumor staging, postoperative therapy, distant metastases, prognosis, and survival [10]. Plenty of factors contribute to disease recurrence and metastasis, among which occult lateral compartment metastasis plays a vital role. So a complete neck LND is of great importance for preventing PTC recurrence and metastasis. If there is preoperative evidence (on exam or ultrasound and neck CT scan) of central or lateral node metastases, therapeutic lymph node dissection would be performed. However, it is still ambiguous whether prophylactic LLND should be performed in a situation in which neither ultrasound nor neck CT scan proves LN metastasis. Researchers are against prophylactic lateral LND due to the wide disturbed surgical field which may cause a series pre- and postoperative complications such as postoperative pain, worsening life quality of patient (wound pain, hoarseness), and injuries to vessels (Chyle leak, hemorrhage) and nerves (accessory, ramus mandibularis, sympathetic (Horner's syndrome), phrenic, brachial plexus, and cutaneous cervical plexus).

Therefore, finding a management which can balance the benefit of disease control by lateral LND and the loss of life quality greatly needs to be addressed. Lee et al. applied preoperative lymphoscintigraphy combined with a hand-held collimated gamma probe to search for “radioactive” lymph nodes to searching for the sentinel lymph node [11]. The tracer they used was a 99mTc-tin colloid which was intratumorally injected under ultrasound guidance. The radical lateral LND was performed if the picked SLN had metastasis proven by frozen pathology. They believed that sentinel lymph node biopsy is a useful method for evaluating the occult lateral neck lymph node status in patients with PTC. This method of detecting SLN was reported by others. Besides radio-guided SLNB (rSLNB), the LN tracer also includes other kinds of dye techniques, such as CN, methylene blue and India ink, and indocyanine green (ICG).

Carbon nanoparticle suspension has good lymph tropism. After injection into the thyroid tissue, it will be absorbed by the lymph system quickly selectively blacking the lymph vessels and nodes, which will enter into the partial lymph pipe network and lymph gland without blood pipe absorption due to its 150 nm diameter which is bigger than the blood capillary gap (about 30-50 nm) and less than the lymphatic capillary gap (100-500 nm). Compared with the first generation of lymph tracers (methylene blue and India ink), CN suspension has the advantages of great dispersivity, with the parathyroid gland not stained and even dying [12]. In comparison with the radioactive tracer, CN is much easier to operate, requires no special equipment, is radiationless, and is safer for the human body. Carbon nanoparticle suspension has been widely employed for lymph node tracing in lots of malignant tumors such as gastric cancer, breast cancer, cervical cancer, oral cancer, colorectal cancer, and lung cancer. Recently, CN was applied in thyroid cancer as reported by several authors. Zhang et al. reported that carbon nanoparticle-assisted sentinel lymph node biopsy can detect incidental thyroid carcinoma which prevents the need for a second surgery [13].

**Figure 3:**
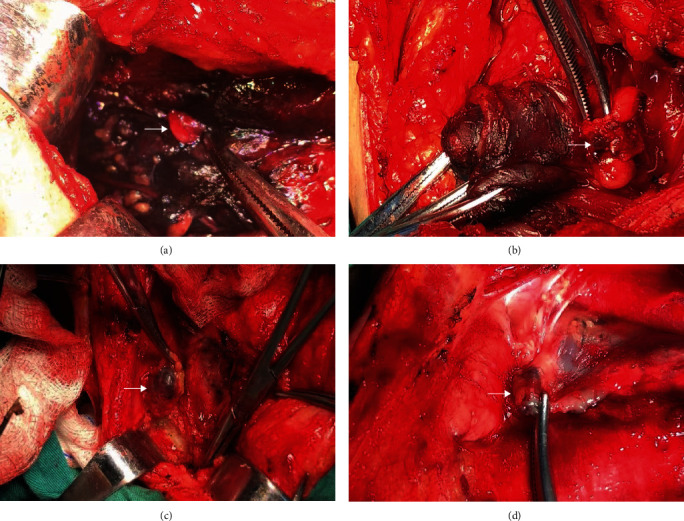
The parathyroid glands were not stained because of sharing different lymphatic systems. Therefore, CN suspensions did help highlight parathyroid glands (a) (→ indicates the unstained parathyroid gland). (b–d) shows the staining of lymph nodes by CN in levels VI (b) and III (c, d). White arrows indicate the black-stained lymph nodes.

Because carbon nanoparticles cannot stain the parathyroid gland, the parathyroid glands can be easily identified and protected to reduce the complications of transient hypoparathyroidism [14] (Figure 3(a)). This feature has great significance especially for those who unfortunately have bilateral thyroid cancer thus with a high risk of damage to the parathyroid gland or for patients who need a second neck surgery due to recurrence and other reasons. In this situation, CN can assist the surgeons to finish the bilateral central compartment dissection and protect the parathyroid gland to the maximum extent at the same time. In our study, none had permanent hypoparathyroidism which indicated that CN can effectively protect the parathyroid gland making CLND safer.

This study has some limitations. First, it showed that lateral neck nodal involvement is not uncommon, at 28%. However, it did not conduct long-term follow-up observations to illustrate the benefits of preventive LLND. Whether the use of CN suspension can improve the long-term prognosis and reduce the recurrence still needs to be explored in the future. Second, the conclusion in this study needs a greater sample clinical study for further confirmation.

In conclusion, this study indicated that lateral LN metastasis in CN0PTC is not uncommon. Thus, CN suspension-assisted identification of drainage LNs should be considered for facilitating the modified resection of lymph nodes at the central and lateral compartments of the neck, reducing neck dissection-associated complications, especially for the exploration and management of the lateral compartment in patients without definite evidence of lateral neck metastasis.

## Figures and Tables

**Table 1:** General information and basic data of CN0PTC patients between CN group and control group.

	CN group 33 cases 40 sides	Control group 28 cases 34 sides	*χ* ^2^(*t*/*Z*) value	*p* value
Age, y (mean ± SD)	43.55 ± 11.56	42.89 ± 14.096	*t* = 0.199	0.338
Gender, M/F			*χ* ^2^ = 0.950	0.330
Male	8	4		
Female	25	24		
Laterality			*χ* ^2^ = 0.000	0.984
Bilateral	7	6		
Multinodules^1^			*χ* ^2^ = 0.728	0.394
Single	27	26		
Multifoci	13	8		
Tumor size (*n*, %)^2^			*χ* ^2^ = 0.022	0.883
*T* ≤ 1 cm	30	26		
1 cm < *T* ≤ 2 cm	10	8		
Tumor site			*χ* ^2^ = 1.774	0.183
Upper pole	25	16		
Middle/lower portion	15	18		
BRAF mutation	26	21	*χ* ^2^ = 0.123	0.726
LN metastasis/side				
Central	2	4	*χ* ^2^ = 3.606	0.058
Lateral	5	—		
Central+lateral	5	—		
Complications				
Unilateral vocal cord palsy	0	2	*χ* ^2^ = 2.437	0.118
Hemorrhage	0	0		—
Wound infection	0	0		—
Seroma	2	1	*χ* ^2^ = 0.201	0.654
Transient hypocalcemia	6	17	*χ* ^2^ = 11.666	0.001^∗^
Transient hypoparathyroidism	9	14	*χ* ^2^ = 3.958	0.047^∗^

^1^Described by side. ^2^Calculated by the maximum diameter if multifocal lesions existed. LN: lymph node. ^∗^*p* < 0.05.

**Table 2:** Distribution of black-stained LNs in the lateral compartment of CN0PTC patients.

	Number of sides	Positive for metastasis proof by intraoperative frozen section
No black-stained LN detected in lateral compartment	11	—
II level	1	0
III level	17	5
II, III level	6	5 (II: 1; III: 2; II+III: 2)
III, IV level	5	0
Total	40	10

PTC: papillary thyroid carcinoma; LN: lymph node.

## Data Availability

We can provide the underlying data if needed.
